# Advocating the role of trained immunity in the pathogenesis of ME/CFS: a mini review

**DOI:** 10.3389/fimmu.2025.1483764

**Published:** 2025-03-25

**Authors:** Bart Humer, Willem A. Dik, Marjan A. Versnel

**Affiliations:** ^1^ Department of Immunology, Erasmus Medical Center, University Medical Center Rotterdam, Rotterdam, Netherlands; ^2^ Laboratory Medical Immunology, Department of Immunology, Erasmus Medical Center, University Medical Center Rotterdam, Rotterdam, Netherlands

**Keywords:** ME/CFS, PASC, trained immunity, innate immunity, epigenetics, metabolomics

## Abstract

Myalgic encephalomyelitis/chronic fatigue syndrome (ME/CFS) is a complex chronic disease of which the underlying (molecular) mechanisms are mostly unknown. An estimated 0.89% of the global population is affected by ME/CFS. Most patients experience a multitude of symptoms that severely affect their lives. These symptoms include post-exertional malaise, chronic fatigue, sleep disorder, impaired cognitive functions, flu-like symptoms, and chronic immune activation. Therapy focusses on symptom management, as there are no drugs available. Approximately 60% of patients develop ME/CFS following an acute infection. Such a preceding infection may induce a state of trained immunity; defined as acquired, nonspecific, immunological memory of innate immune cells. Trained immune cells undergo long term epigenetic reprogramming, which leads to changes in chromatin accessibility, metabolism, and results in a hyperresponsive phenotype. Initially, trained immunity has only been demonstrated in peripheral blood monocytes and macrophages. However, more recent findings indicate that hematopoietic stem cells in the bone marrow are required for long-term persistence of trained immunity. While trained immunity is beneficial to combat infections, a disproportionate response may cause disease. We hypothesize that pronounced hyperresponsiveness of innate immune cells to stimuli could account for the aberrant activation of various immune pathways, thereby contributing to the pathophysiology of ME/CFS. In this mini review, we elaborate on the concept of trained immunity as a factor involved in the pathogenesis of ME/CFS by presenting evidence from other post-infectious diseases with symptoms that closely resemble those of ME/CFS.

## Graphical Abstract

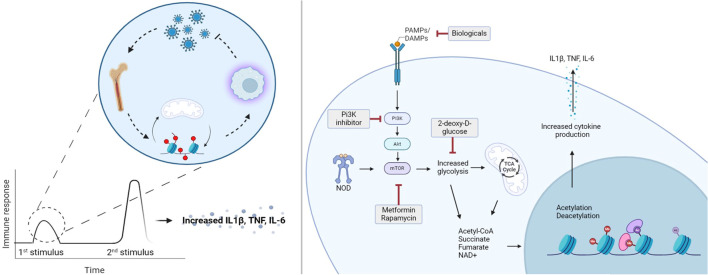

## Introduction

Myalgic encephalomyelitis/chronic fatigue syndrome (ME/CFS) is a complicated chronic disease of which the pathogenesis is largely unknown. According to the Center for Disease Control (CDC), 836,00 to 2.5 million Americans were diagnosed with ME/CFS in 2015 (1). While, estimating global prevalence is challenging since many countries do not recognize ME/CFS, it is thought to affect 0.89% of the world population (1). ME/CFS occurs in all racial and ethnic groups but is ~2 times more prevalent in women (2). Although the prevalence is highest in individuals aged between 40-60 years, ME/CFS can develop in all age groups (2). In the absence of diagnostic laboratory markers, diagnosis relies entirely on symptom questionnaires of which the Canadian Consensus Criteria (CCC) and Fukuda definition are used most often (1). A universally accepted diagnostic consensus does not exist, which has led to multiple definitions of ME/CFS in both research and clinical practice (3). Although diagnostic criteria are dispersed among different definitions, all cases of ME coexist with CFS. The symptoms of ME/CFS are diverse and often overlap with those of other diseases, making the diagnostic process lengthy and challenging. Clinical symptoms reported in ME/CFS patients include extreme fatigue, post-exertional malaise (PEM), muscle pain, sleep impairment, cognitive dysfunction, flu-like symptoms, sensory intolerance, gastrointestinal and genitourinary problems, severely impacting quality of life (3). Although these symptoms are often chronic, disease activity can fluctuate, resulting in varying disease prognoses among patients (3, 4). Since the pathophysiology still is unknown, no drugs for treatment are available. Therefore, current treatment of ME/CFS primarily focuses on symptom management, with particular emphasis on preventing exacerbation of PEM (4).

Although ME/CFS is highly variable in its presentation, ~60% of patients report an infectious period before developing the condition (5). This preceding infection leads ME/CFS patients to report persistent “sickness behavior” (SB), encompassing symptoms common to chronic fatigue syndromes, such as fever, myalgia, and general fatigue (6). SB is considered an evolutionary trait to cope with the active infection. In case of infection, activation of inflammatory pathways causes a cytokine response, resulting in energy consumption for survival tasks, which allows the individual to cope with the infection (6). When the cytokine release response becomes chronic, SB persists. This suggests that the initial infection activates a cascade of events that contributes to keeping this SB state, despite the infection being resolved. In ME/CFS, elevation of a wide range of cytokines, including CXCL10, IFN-γ, IL-4, IL-5 and IL-7, was reported and shown to be correlated with disease severity (7, 8).

Interestingly, the recent COVID-19 pandemic has led to a rise in new cases of ME/CFS. An estimated 60% of post-acute sequelae of COVID-19 (PASC), formerly referred to as long COVID, fulfills ME/CFS criteria (9). This supports the hypothesis that a preceding infection triggers the development of chronic fatigue (10). Therefore, research aimed at understanding the pathogenesis of PASC can provide valuable insights into the mechanisms underlying ME/CFS. Here, we propose that trained immunity, also referred to as innate immune memory, may contribute to the pathogenesis of ME/CFS. In this review, we describe the concept of trained immunity, and present evidence supporting its involvement in conditions with symptoms resembling those of ME/CFS. The purpose of this review is to enlighten the perspective of trained immunity as a contributing factor to the pathogenesis of ME/CFS.

## Trained immunity

Trained immunity is defined as the acquisition of immunological memory by innate immune cells (11, 12). Once innate immune cells have been exposed to activating stimuli, their response to subsequent stimuli is adjusted, leading to hyperresponsive phenotypes (12). Importantly in trained immunity, the secondary stimuli that induces the trained response can be unrelated to the first stimulus, which makes the distinction from adaptive immune memory.

Studies on trained immunity in mice showed that one month after elimination of an influenza infection, re-infection of the mice with *S. pneumoniae* resulted in increased production of IL-6 (13). The initial influenza encounter induced epigenetic changes required for cytokine production during the subsequent response to S. *pneumoniae*, thereby establishing innate immune memory (13). Thus, trained innate cells undergo long-lasting epigenetic alterations that result in a shift from homeostatic baseline towards a more hyperresponsive phenotype. Additional evidence for trained immunity *in vivo* was shown upon administration of Bacillus Calmette-Guérin (BCG) vaccine to mice lacking an adaptive immune system conferred cross protection when reinfected with *Candida albicans* (14). Additionally, PBMCs from humans showed increased cytokine production after BCG vaccination when exposed to *S.aureus, and C.albicans* (14), suggesting cross protection in humans as well (14). Besides exogenous ligands, endogenous ligands and cytokines are also capable of inducing immunological memory in innate immune cells. Examples of such endogenous stimuli are lipoprotein(a), oxidized low density lipoproteins, aldosterone, catecholamines, cytokines, and uric acid (15–18). This data suggests that also naturally occurring substances may invoke the secondary response in trained cells.

## Epigenetic factors leading to trained immunity

Accessibility of gene transcription is determined by histone acetyltransferase and histone deacetylases which can modify histone configuration by methylation, acetylation, phosphorylation and ubiquitination (19–21). Upon an infection, proinflammatory stimuli initiate acetylation of histones, resulting in more accessible chromatin structures at gene promoter sites, leading to increased accessibility and therefore increased transcription. In trained immunity, after a first trigger, the accessibility of promoter regions in chromatin is more sensitive for subsequent stimuli, that convert the cell towards a hyperresponsive state (19, 22). Upon encountering a second stimulus, gene transcription occurs more readily, leading to the enhanced response observed in trained immune cells, including monocytes, macrophages, dendritic cells, natural killer cells, and innate lymphoid cells (11). While not specific for trained immunity, studies that investigated epigenetic changes in ME/CFS have found alterations in immune cells and evidence of metabolic reprogramming (23, 24). While epigenetic alterations play a critical role in the development of trained immunity, it functions in concert with metabolic changes.

## Metabolic rewiring

Immune cells in a homeostatic state exhibit low energy consumption, primarily deriving their energy from oxidative phosphorylation and fatty acid oxidation (25). During inflammatory activation states energy demands increase, causing a shift from oxidative phosphorylation to glycolysis to meet this heightened demand (25). Besides providing rapid energy production, metabolic intermediates from glycolysis regulate epigenetic changes involved in trained immunity (19). Thus, both metabolic and epigenetic processes form a joint system essential for the development of trained immunity.

Following training with cell wall components of *Candida albicans*, monocytes exhibited increased glucose consumption with an increase of glycolysis by-products (26). Simultaneously, gene expression analysis revealed that the glycolysis regulator, mTOR, and mTOR-related genes were upregulated in comparison with untrained monocytes (26). Inhibitors of mTOR, such as rapamycin and metformin, were able to interrupt the development of trained immunity when administered during training with various stimuli including BCG vaccination (26, 27). These studies indicate that metabolic rewiring is essential to induce trained immunity, as it provides energy demands and intermediates necessary for epigenetic adaptations to occur. Thus, both metabolism and epigenetic regulation can be manipulated to prevent development of trained immunity.

## Longevity of trained immunity

Innate immune cells have a limited lifespan (28, 29), suggesting that trained immunity is engraved deeper within the hematopoietic system. Studies investigating this have shown that long-term effects of trained immunity are retained by changes in hematopoietic stem cells in the bone marrow (30). Intravenous BCG vaccination of mice was found to lead to enhanced expansion of bone marrow derived hematopoietic stem cells (HSC), mesenchymal stem cells and increased myelopoiesis as opposed to lymphopoiesis (30). When bone marrow of BCG vaccinated mice was transferred into T-cell depleted mice these transplanted mice were more efficient at controlling *Mycobacterium tuberculosis* (*Mtb)* infection (30). These data show that descendant immune cells from bone marrow of BCG vaccinated mice remained effective at killing *Mtb*. In addition, it was shown that HSC from BCG vaccinated humans displayed different transcriptomic profiles (31). Interestingly, transcriptome differences persisted in peripheral blood monocytes up to at least 3 months after BCG vaccination. Another study indicates long term trained immunity after a BCG vaccination in monocytes of children for over a year (32). Despite the limited number of studies that explored HSC in relation to trained immunity, current data suggests that alterations in HSC are inherited by daughter cells, that subsequently contribute to long term persistence of trained immunity. While these findings help explain the chronic nature of most post-infectious syndromes, further research is required to explore trained immunity over longer timescales.

## Trained immunity in post-infectious and autoimmune diseases

Evidence regarding trained immunity in specifically ME/CFS is still lacking. However, a few studies investigated features of trained immunity in recovered hospitalized COVID-19 patients, months after discharge (33–35). Here they observed increased monocyte counts and proinflammatory transcriptomic profiles in comparison with healthy controls (33–35). Epigenetic analysis revealed that these monocytes had increased chromatin accessibility for genes responsible for cell migration and production of inflammatory mediators (34). These data suggest that monocytes were skewed towards a pro-inflammatory phenotype, which was corroborated by PBMCs from convalescing COVID patients producing more IL-1β and IL-6 when stimulated with spike-nCoV pseudovirus (34).

Long term trained immunity has been studied in hematopoietic stem and progenitor cells (HSPC) of convalescing COVID-19 patients, up to a year after recovery (35). Epigenetic and transcriptomic analysis reveal increased activation of genes for HSPC to differentiate into myeloid cells, as opposed to other leukocyte lineages (34, 35). In addition, the prevalence of granulocyte-monocyte progenitors was significantly higher, which corresponds with the transcriptomic profile that skews HSPC towards myeloid development (35). Interestingly, the chromatin accessibility signatures in HSPC and monocytes, displayed overlapping epigenetic and transcriptional programs. These data suggest persistent post-infectious transcriptional and epigenetic programs in HSPC, which are maintained in descending monocyte lineages (35). These studies indicate that exposure of the immune system to SARS-CoV-2 can engrave a trained phenotype in multiple cell lineages of the hematopoietic system. While such studies have not been performed for ME/CFS, transcriptional monocytes dysregulation and heightened serum cytokines correlating with disease severity have been described (7, 36).

Q-fever, is another post-infectious disease with chronic fatigue as a hallmark in those with long lasting symptom burden. Approximately 40% of those infected with *Coxiella burnetti* develop symptoms, resembling flu-like illness accompanied by fever with pneumonia or hepatitis (37). Interestingly, an estimated 20% of patients with acute Q fever develop a long lasting post-infectious fatigue syndrome with symptoms that overlap with those observed in ME/CFS (38). Monocytes of acute Q fever patients had increased production of IL-1β, IL-1Ra, IL-6 and IL-10 up to 27 days after their last sick day (39). IL-10 levels remained elevated at six months after the acute Q-fever infection. This suggests occurrence of long term alterations of monocytes and macrophages and aligns with a trained phenotype (39).

Primary Sjögren’s disease (pSjD) is a systemic autoimmune disease with sicca symptoms as a hallmark and with 70% of patients having debilitating fatigue. Subgroups of ME/CFS also present with autoantibodies against nuclear and membrane structures, have similar sicca symptoms as observed in pSjD and fulfill diagnostic criteria for pSjD (40, 41). Previously, we have shown that activation of the type I interferon (IFN-I) pathway in monocytes of pSjD was associated with higher clinical disease activity scores (42). Subsequently, we linked this IFN-I activity to trained immunity by demonstrating that IFN-I functions as a potent trainer for monocytes (18, 43). In an *in vitro* model in THP-1 cells, training with IFN-I induced elevated production of IL-6, TNFα and CCL2 and increased glucose consumption upon LPS restimulation (18). These data suggest that IFN-I induces a trained immunity phenotype in pSjD and possibly other disease conditions.

## Pharmacological inhibition

Effectively trained immunity consists of two main pillars: the initial training, and the secondary stimulus. While post-infectious diseases are instigated by the initial infectious agent, which can be regarded as the first training, the hyperresponsive result will only occur when a second stimulus is encountered. Treatment targets are primarily those that are altered after the initial training, meaning that drugs that modify the epigenetic and/or metabolic changes are of interest. Many studies regarding pharmacological inhibition of trained immunity, with mTOR as primary target, are performed *in vitro*. The mTOR inhibitor metformin has been extensively used to block trained immunity *in vitro* and was recently tested *in vivo* in PASC (23). In this study metformin use was investigated whether it could reduce PASC incidence (44). Strikingly, outpatient metformin treatment reduced PASC incidence by 41% (44). Another mTOR inhibitor, rapamycin, is currently being investigated in an observational clinical trial for reduction of overall symptom burden, and whether it improves quality of life in ME/CFS (NCT06257420). Although these studies were not initiated to retaliate trained immunity *in vivo*, the results support a role for therapeutics if trained immunity is involved in ME/CFS.

## Conclusion and future directions

In this review, we discussed the complexities of ME/CFS and propose a role for trained immunity based on other conditions with chronic fatigue and/or a post infectious nature. Current treatment methodologies are primarily based on cognitive behavioral therapy. Effective pharmacological management does not exist. The emergence of COVID-19 has regained interest in post-infectious syndromes, with novel concepts for the cause and persistence of these conditions to explain the symptoms and identify novel treatment targets. Here we propose a role for trained immunity in ME/CFS by reviewing the current available evidence for this hypothesis. Especially studies in post infectious conditions, where an initial infection provokes some of the instigators required for trained immunity development support such a hypothesis. In summary, the initial infection causes a swift change in epigenetics and metabolism resulting in a hypersensitive and hyperresponsive phenotype after a second exposure to a pathogen. After an acute infection, long term changes are induced by epigenetic and metabolic changes in the hematopoietic stem cell system. The descendants from these stem cells are innate myeloid cells equipped with a more proinflammatory phenotype. This increased sensitivity towards continuous, naturally occurring stimuli could provoke sustained inflammatory activity instigating cytokine release leading to sickness behavior, resulting in the chronic fatigue these patients have.

While ME/CFS is a heterogenous disease in all aspects, the initial infection can act as an important marker to identify different pathogenetic mechanisms and subgroup patients based upon this feature. Future studies should explore post infectious ME/CFS by studying factors responsible for trained immunity such as epigenetic modifications, metabolic pathways, and infections. These studies will form a basis to further subgroup patients for development of novel biomarkers for diagnosis, and targeted treatment.
